# Isolation, Identification, and Diversity Analysis of Endophytic Fungi from Sweetpotato

**DOI:** 10.3390/jof12060394

**Published:** 2026-05-29

**Authors:** Shi-Xin Wang, Jing Li, Xin-Xin Zhang, Han-Hong Xu, Li-Fei Huang

**Affiliations:** 1 State Key Laboratory of Green Pesticide, College of Plant Protection, South China Agricultural University, Guangzhou 510642, China; 17513263605@163.com; 2 Crops Research Institute, Guangdong Academy of Agricultural Sciences, Guangzhou 510640, China; 13213163605@163.com (J.L.); zhangxinxinatda@163.com (X.-X.Z.); 3 Shanwei Agricultural Science Academy, Shanwei 516600, China

**Keywords:** endophytic fungi, sweetpotato, culturable fungal resource, diversity, biocontrol potential

## Abstract

Endophytic fungi contribute substantially to plant health, yet the diversity, community composition, and tissue distribution of culturable fungal endophytes in sweetpotato remain poorly characterized. In this study, endophytic fungi were isolated from roots, old stems, tender stems, petioles, and leaves of the scab-resistant leafy variety ‘Guangcaishu 16–19’, the scab-susceptible leafy variety ‘Guangcaishu No. 5’, and the tuberous variety ‘Guangshu 87’ were identified based on morphological characteristics and ITS sequencing. ITS sequencing identified 492 fungal isolates belonging to 63 putative taxa in 31 genera. *Colletotrichum* was the dominant genus in the leafy varieties, whereas *Chaetomium* was dominant in the tuberous variety. The isolated endophytic fungi were widely distributed and tissue-specific, with genus-level distribution following the pattern “leaves and stems > roots”. Alpha diversity followed the order ‘Guangcaishu 16–19’ > ‘Guangcaishu No. 5’ > ‘Guangshu 87’. The fungal communities of the leafy varieties were the most similar, and their root-associated communities exhibited higher alpha diversity than those of the tuberous variety. In addition, scab-resistant varieties exhibited higher endophytic fungal diversity. Overall, endophytic fungal communities in sweetpotato exhibited high alpha diversity, and significant differences in community structure were observed among variety tissues. These findings provide culturable fungal resources for future screening of beneficial endophytic fungi, bioactive metabolites, and potential biocontrol agents.

## 1. Introduction

Sweetpotato (*Ipomoea batatas* (L.) Lam.) is a perennial herbaceous plant belonging to the family Convolvulaceae and is an important crop used for food, feed, and industrial purposes [1,2,3]. The sweetpotato stems and leaves are rich in protein and amino acids [4,5] and possess antioxidant, anti-cancer, and other biological activities [6]. Their content of relatively high soluble dietary fiber may also contribute to beneficial metabolic effects, including reduced blood glucose, serum lipid, and levels of hepatic cholesterol [7]. In addition, the sweetpotato tubers are rich in starch [8], soluble sugars, and reducing sugars [9], and are widely used in food processing and related industries [10]. While these traits are primarily determined by plant genetic and physiological factors, accumulating studies indicate that endophytes colonizing sweetpotato tissues also play important roles in plant growth, metabolism, and the formation of traits.

Over the course of evolution, plants have developed symbiotic relationships with diverse microorganisms, including bacteria, fungi, archaea, algae, and protists. These microorganisms collectively constitute the plant-associated microbiome [11]. Endophytes interact with plants and can profoundly affect the survival, evolution, adaptability, and stress resistance of plants [12]. In particular, the beneficial effects of endophytic fungi, which are widely present within plant tissues, have been extensively documented [13]. These beneficial effects are primarily reflected in three aspects. First, the endophytic fungi can promote plant growth by producing phytohormones, such as auxin and gibberellin, or by secreting phosphatases and siderophores that enhance host nutrient uptake and thereby increase the plant biomass [14,15]. Secondly, they can enhance host resistance to both biotic stresses, such as pathogen infection, and abiotic stresses, such as drought and salinity [16,17]. Third, endophytic fungi can produce diverse secondary metabolites with considerable potential for pharmaceutical development and biological control [18,19]. For example, *Taxomyces andreanae*, an endophytic fungus isolated from *Taxus brevifolia*, can produce paclitaxel, a well-known anticancer drug [20]. However, the relationship between endophytes and their hosts is not fixed, and its outcomes depend on the specific combination of microbial strains, host tissues, and environmental conditions. Under certain conditions, endophytic fungi that are initially symbiotic may shift to pathogenic strains and infect their hosts [21]. For example, *Fusarium oxysporum* f. sp. *cubense* may persist asymptomatically in some banana varieties, but in susceptible hosts, such as Cavendish bananas (*Musa acuminata* Cavendish Subgroup), particularly under environmental stress, it can cause lethal *Fusarium* wilt and pose a major threat to banana production [22]. Therefore, endophytes can exert both beneficial and detrimental effects on plants, highlighting their dual roles as mutualists and potential pathogens.

Beneficial endophytic microorganisms associated with sweetpotato provide important biological resources to improve the yield and stress tolerance. Previous studies have begun to reveal the functional roles of endophytes in sweetpotato. For example, Pei et al. [23] reported that arbuscular mycorrhizal fungi can promote the growth of sweetpotato and enhance stress tolerance. Huang et al. [24] isolated 17 endophytic bacterial strains belonging to 10 genera from sweetpotato and systematically characterized their growth traits, phosphate-solubilizing ability, and antibacterial activity. Indrawati et al. [25] isolated and identified 14 endophytic bacterial strains from multiple varieties of sweetpotato and studied the relationship between their metabolites and antibacterial properties. Marques et al. [26] showed that the endophytic bacterial communities in the sweetpotato tubers are primarily shaped by the plant growth stage, while the host genotype primarily exerts a detectable effect at the early growth stage. Dhungana et al. [27] isolated eight endophytic bacterial strains from sweetpotato and discovered that two could fix nitrogen (N) and biosynthesize the phytohormone indole-3-acetic acid (IAA) to promote the growth of plants. Khan et al. [28] also identified 11 endophytic bacteria from sweetpotatoes and confirmed that some strains produce IAA and can fix N. Although these studies have provided preliminary insights into the specific functions of endophytes in sweetpotatoes, the current studies have primarily focused on bacterial groups or screening strains with specific functions. In contrast, the diversity, community composition, and tissue distribution of culturable endophytic fungi in different sweetpotato varieties remain insufficiently investigated. Although culture-based isolation is a conventional method, it remains essential for obtaining viable fungal isolates for functional assays and biocontrol screening. Therefore, this study aimed to characterize the diversity, community composition, and tissue distribution of culturable endophytic fungi in two leafy sweetpotato varieties with contrasting responses to scab disease and one tuberous variety. These findings provide a collection of culturable fungal isolates and support future screening of strains with potential roles in growth promotion, disease resistance, and bioactive metabolite production.

## 2. Materials and Methods

### 2.1. Study Site, Plant Materials, and Sample Collection

This study was conducted at the Zhongluotan Baiyun Base of the Guangdong Academy of Agricultural Sciences, Guangzhou, China (113.44° E, 23.39° N). The region features a subtropical monsoon climate. The soil is sandy loam with pH of 6.74, organic matter 1.35%, hydrolyzable N 75.6 mg kg^−1^, effective phosphorus 128 mg kg^−1^, and quick-acting potassium 180 mg kg^−1^. The experimental area was managed under standard agronomic practices, with rice planted in the first half of the year and sweetpotatoes in the second half. After planting, all experimental plots received identical pesticide treatments under uniform field management conditions. Specifically, 40 mL per mu (1 mu ≈ 666.7 m^2^) of 8.8% quizalofop-P-ethyl herbicide was applied for weed control, and 2000 g per mu of 5% cyfluthrin-phoxim insecticide was applied for sweetpotato weevil control. The interval between pesticide application and sample collection was 45 days, and no other pesticides were applied during this period.

**Figure 1:**
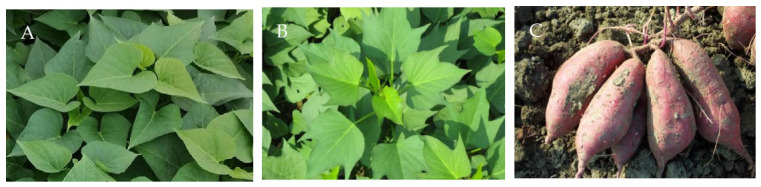
Three varieties of sweetpotato. (**A**–**C**): ‘Guangcaishu 16–19’ (**A**), ‘Guangcaishu No. 5’ (**B**) and ‘Guangshu 87’ (**C**).

The test materials were as follows (Figure 1). Three sweetpotato varieties bred by the Crop Research Institute of the Guangdong Academy of Agricultural Sciences (Guangzhou, China) were selected. Of the three varieties, two were leafy varieties, whereas the other was a tuberous variety. Among these, the varieties grown for their leaves included ‘Guangcaishu 16–19’, which is resistant to scab, and ‘Guangcaishu No. 5’, which is susceptible to this pathogen. The tuberous variety was ‘Guangshu 87’. Each variety was sampled in three biological replicates, and three plants were selected from each replicate. All selected plants were free of visible disease symptoms, insect damage, and mechanical injury, and exhibited comparable vine vigor, leaf expansion, and overall growth status. Entire plants were carefully dug out, and the adhering soil was gently removed from the roots. Samples were collected 45 days after planting, when the plants had reached the vegetative growth stage. Tissues around the sixth to seventh fully expanded leaves were selected for sampling. The roots, stems, leaves, and petioles were sampled separately, labeled individually, and transported to the laboratory on ice. The residual soil was gently rinsed off with tap water, and the samples were then transferred to a laminar flow hood for tissue separation.

### 2.2. Isolation of the Endophytic Fungi

**Figure 2:**
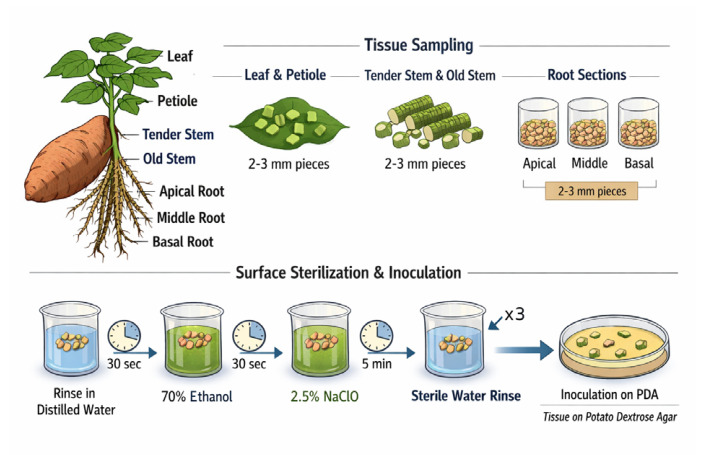
Procedures to isolate the endophytic fungi.

Tissue sections were cut from different parts of the sweetpotatoes to isolate the endophytic fungi (Figure 2). The leaf and petiole tissues were cut into 2–3 mm segments. Stem tissues were collected from the third to fourth node at the apex for the tender stems and near the ground level for the old stems. Both types of stems were cut into 2–3 mm stem segments. A total of 2–3 mm pieces of the root tissues were sampled from the apical, middle, and basal regions. A total of 540 tissue fragments were obtained across all varieties and tissues.

The tissues were surface-sterilized as described by Schulz et al. [29], with minor modifications. Briefly, the tissue segments were washed with distilled water for 30 s, immersed in 70% ethanol for 30 s, treated with 2.5% sodium hypochlorite (NaClO) for 5 min, and then rinsed three times with distilled water. After they were dried on sterile filter paper, the tissue segments were placed on potato dextrose agar (PDA) plates with the cut surface facing downward. A blank control was included in each batch by spreading 100 μL of the final rinse water onto a PDA plate. Control plates were incubated under the same conditions used for fungal isolation, and surface sterilization was considered effective when no fungal or bacterial growth was observed after five days.

### 2.3. Endophytic Fungal Culture

The inoculated Petri dishes were incubated in a constant-temperature incubator at 25 °C under a 12 h light/12 h dark cycle. Once new mycelia grew around the tissue segments, mycelia from the colony margins were transferred to fresh PDA plates. This procedure was repeated to produce a purified strain.

### 2.4. Endophytic Fungal DNA Extraction and PCR Amplification

Genomic DNA was extracted from sweetpotato endophytic fungal isolates using an Ezup Column Fungi Genomic DNA Purification Kit (Cat. No. B518259-0100; Sangon Biotech, Shanghai, China) and used as the template for PCR amplification. The universal primers ITS1 (5′-TCCGTAGGTGAACCTGCGG-3′) and ITS4 (5′-TCCTCCGCTTATTGATATGC-3′) were utilized to amplify the ITS region of the fungal strains. The PCR reaction volume was 50 μL and included 5 μL 10 × PCR buffer, 4 μL dNTPs (2.5 mmol/L each; final concentration, 0.2 mmol/L each), 4 μL MgCl_2_ (25 mmol/L stock; 2.0 mmol/L final), 1 μL each of ITS1 and ITS4 primers (10 μmol/L stock; 0.2 μmol/L final), 0.5 μL Taq DNA polymerase (5 U/μL; 2.5 U per reaction), and 1 μL DNA template. ddH_2_O was added to bring the volume to 50 μL. The amplification program was as follows: initial denaturation at 95 °C for 3 min, 35 cycles of 94 °C denaturation for 40 s, 52 °C annealing for 50 s, and 72 °C extension for 1 min, followed by a final extension at 72 °C for 10 min. The amplified products were sequenced by Shanghai Majorbio Bio-pharm Technology Co., Ltd. (Shanghai, China).

### 2.5. Sequence Analysis and Construction of the Phylogenetic Tree

The sequences obtained were compared with the reference sequences in the NCBI database using BLAST (https://blast.ncbi.nlm.nih.gov/Blast.cgi, 23 September 2019). Phylogenetic trees were subsequently constructed using the neighbor-joining method in MEGA 7, with evolutionary distances estimated using the p-distance model and branch support assessed with 1000 bootstrap replicates. The taxonomic identity of each strain was determined by combining the phylogenetic analysis with morphological characteristics. The sequence data have been deposited in the NCBI GenBank database under accession numbers PZ296779–PZ297270. Detailed information for each sequence is provided in Table S1.

### 2.6. Isolation Frequency and Diversity Analysis

The separation frequency and diversity index for each kind of tissue were calculated using the corresponding formulae. All the data were analyzed using Microsoft Excel (2025) (Redmond, WA, USA). The isolation frequency reflects the occurrence of a species in the samples, whereas the relative isolation frequency reflects the proportion of isolates among the total isolates.

Isolation Frequency:
  (1) $$IR=\frac{\mathrm{Number}\;\mathrm{of}\;\mathrm{tissue}\;\mathrm{blocks}\;\mathrm{yielding}\;\mathrm{fungal}\;\mathrm{isolates}}{\mathrm{Total}\;\mathrm{number}\;\mathrm{of}\;\mathrm{tissue}\;\mathrm{blocks}}\times 100\%\;\;\;$$

Relative Isolation Frequency:
  (2) $$RF=\frac{\mathrm{Number}\;\mathrm{of}\;\mathrm{isolates}\;\mathrm{belonging}\;\mathrm{to}\;a\;\mathrm{given}\;\mathrm{genus}}{\mathrm{Total}\;\mathrm{number}\;\mathrm{of}\;\mathrm{isolates}}\times 100\%\;\;$$

The alpha diversity was assessed using the Shannon–Wiener, Simpson, and Pielou’s evenness indices. The Shannon and Simpson (typically reported as 1-*D*) indices reflect the community diversity. Higher values indicate greater diversity, whereas the Pielou’s index reflects the evenness of species relative abundances. Higher values indicate a more balanced community.

The specific formula used to calculate the Shannon–Wiener index is shown below:

(3) $$H^{\prime }=-\sum_{i=1}^{k}P_{i}\times \ln P_{i}\;\;\;$$

where *P_i_* is the proportion of isolates belonging to the *i*th genus among the total isolates, and *k* is the total number of endophytic fungal genera isolated from a given plant or tissue sample.

The specific formula to calculate the Simpson Diversity Index is shown below:

(4) $$D=1-{\sum_{i=1}^{k}\left(\frac{N_{i}}{N}\right)}^{2}\;\;\;\;\;$$

where *N_i_* is the number of isolates belonging to the *i*th endophytic fungal genus, *N* is the total number of isolates in a given plant or tissue sample, and *K* is the total number of endophytic fungal genera.

The specific formula to calculate the Pielou’s evenness index is shown below:

(5) $$E=\frac{H^{\prime }}{{H^{\prime }}_{max}}\left({H^{\prime }}_{max}=\ln N\right)\;$$

where *H*′ refers to the Shannon–Wiener diversity index of endophytic fungi in a given plant or tissue sample, *N* is the total number of endophytic fungal genera, and *H*′*_max_* is the maximum diversity index.

The similarity coefficient (*CS*) was used to compare the similarity of endophytic fungal species between two plant or tissue samples. This coefficient is calculated based on the Sorenson coefficient formula, with the specific calculation formula shown below:

(6) CS=2j/(a+b)

where *j* denotes the number of fungal species shared between the two plant or tissue samples, and *a* and *b* represent the total numbers of fungal species recorded in the first and second samples, respectively.

### 2.7. Use of Generative Artificial Intelligence

ChatGPT (OpenAI, San Francisco, CA, USA; GPT-5.5 Thinking; accessed on 8 March 2026) was used solely to improve English-language expression, grammar, and academic wording, and to assist with the preparation of Figure 2 based on content and instructions provided by the authors. It was not used to generate experimental data, perform statistical analyses, conduct taxonomic identification, or draw scientific conclusions. All AI-assisted outputs were reviewed, revised, and approved by the authors.

## 3. Results

### 3.1. Morphological Identification of the Endophytic Fungi in Sweetpotatoes

A total of 513 fungal strains were isolated from different tissues of three sweetpotato varieties. A total of 436 strains were morphologically identified based on colony morphology, texture, color, and growth rate, and their taxonomic placement was verified according to Index Fungorum/Species Fungorum and MycoBank. These endophytic fungi were classified into the phylum Ascomycota and were composed of 3 classes, 8 orders, 10 families, and 13 genera (Table 1).

### 3.2. Molecular Identification of the Endophytic Fungi in Sweetpotato

**Figure 3:**
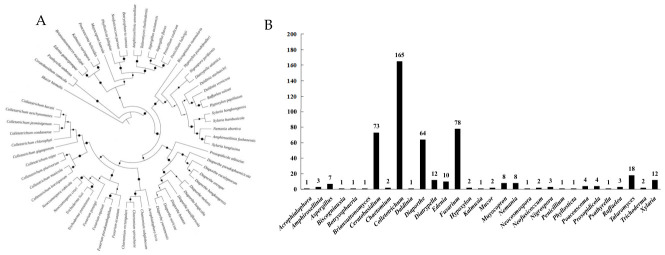
Molecular identification of 492 fungal isolates based on ITS sequences. (**A**): Phylogenetic tree of the endophytic fungi constructed using the neighbor-joining method based on the ITS sequences; (**B**): number of endophytic fungi isolated at the genus level. ITS, internal transcribed sequences.

Molecular analysis generated high-quality ITS sequences for 492 endophytic fungal isolates, which were assigned to 63 putative ITS-based taxa. The remaining 21 isolates were excluded because of failed amplification or low-quality sequencing. A phylogenetic tree was constructed to infer the relationships among these 63 taxa (Figure 3A). These taxa were assigned to 31 genera, including *Acrophialophora*, *Aspergillus*, *Amphirosellinia*, *Biscogniauxia*, *Briansuttonomyces*, *Botryosphaeria*, *Chaetomium*, *Ceratobasidium*, *Colletotrichum*, *Diatrypella*, *Diaporthe*, *Daldinia*, *Edenia*, *Fusarium*, *Hypoxylon*, *Kalmusia*, *Mucor*, *Muyocopron*, *Nemania*, *Neocosmospora*, *Phyllosticta*, *Neofusicoccum*, *Nigrospora*, *Penicillium*, *Poaceascoma*, *Prosopidicola*, *Psathyrella*, *Raffaelea*, *Talaromyces*, *Trichoderma,* and *Xylaria*. Most isolates belonged to Ascomycota, whereas *Ceratobasidium* and *Psathyrella* were assigned to Basidiomycota, and *Mucor* was assigned to Mucoromycota. This suggests that, in the present study, ITS-based molecular identification revealed a broader taxonomic range than identification based solely on morphological characteristics. At the genus level, *Colletotrichum*, *Fusarium*, *Chaetomium*, and *Diaporthe* were the dominant endophytic fungi isolated from the sweetpotato samples. Among these, the genus *Colletotrichum* was the most frequently isolated, accounting for 33.47% of all endophytic fungal strains, followed by *Fusarium* spp. (15.82%), *Chaetomium* spp. (14.81%), and *Diaporthe* spp. (12.98%) (Figure 3B).

### 3.3. Distribution of Endophytic Fungi Among Three Sweetpotato Varieties

**Figure 4:**
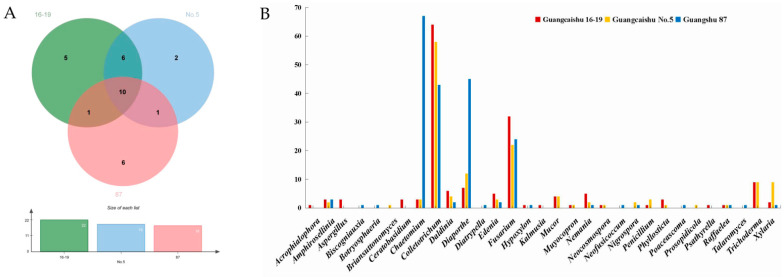
Endophytic fungi isolated from three sweetpotato varieties. (**A**): Distribution of endophytic fungal genera among the three varieties (Venn diagram). (**B**): Number of endophytic fungal isolates at the genus level across the three varieties.

The Venn diagram analysis revealed that the number of endophytic fungal genera followed the order ‘Guangcaishu 16–19’ > ‘Guangcaishu No. 5’ > ‘Guangshu 87’ (Figure 4A). A total of 10 genera were shared among all three varieties. ‘Guangcaishu 16–19’ and ‘Guangcaishu No. 5’ shared six genera, whereas each of these two varieties shared only one genus with ‘Guangshu 87’.

All three sweetpotato varieties harbored diverse endophytic fungi. However, the abundance of isolates within the same genus varied among varieties. At the genus level, *Colletotrichum* and *Fusarium* were the dominant genera in both ‘Guangcaishu 16–19’ and ‘Guangcaishu No. 5’, whereas *Chaetomium*, *Diaporthe*, and *Colletotrichum* predominated in ‘Guangshu 87’ (Figure 4B).

**Figure 5:**
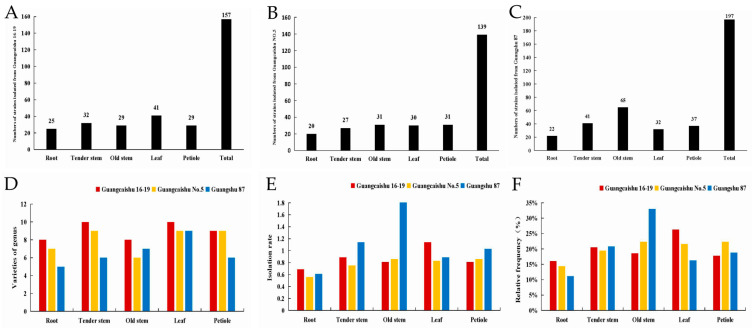
Composition and distribution of endophytic fungi in different tissues of three sweetpotato varieties. (**A**–**C**): Numbers of endophytic fungal isolates recovered from the different tissues of ‘Guangcaishu 16–19’ (**A**), ‘Guangcaishu No. 5’ (**B**), and ‘Guangshu 87’ (**C**). (**D**–**F**): Number of endophytic fungal genera (**D**), isolation frequency (**E**), and relative isolation frequency (**F**) across the different tissue types.

The genus-level distribution of endophytic fungi varied among tissues and sweetpotato varieties (Figure 5). In ‘Guangcaishu 16–19’, the endophytic fungi in the leaves and tender stems had the highest number of genera, number of isolates, isolation frequency, and relative isolation frequency, while the roots showed the opposite trend. The most diverse fungal genera were found in the leaves and petioles of ‘Guangcaishu No. 5’, while the fewest genera were present in the old stems. The separation rate and relative separation frequency were the highest in petioles and old stems, respectively, whereas the root system exhibited the lowest number of isolates, separation rate, and relative separation frequency. ‘Guangshu 87’ exhibited the greatest types of leaves, while the old stems showed the highest number of genera, isolation frequency, and relative isolation frequency. Conversely, the roots displayed the lowest levels of number of isolates, genera, isolation frequency, and relative isolation frequency. Overall, the abundance and genus richness of endophytic fungi at the genus level, as well as their isolation frequency and relative isolation frequency, were higher in the stems and leaves of the three varieties of sweetpotatoes than in their roots.

### 3.4. Characteristics of the Distribution of Endophytic Fungi at the Genus Level in Different Tissues of Three Varieties of Sweetpotato

**Figure 6:**
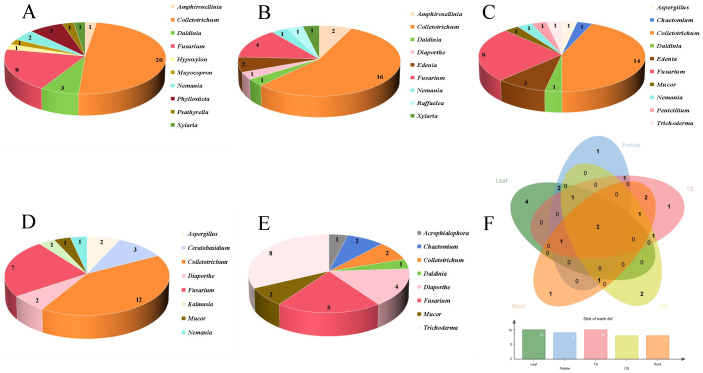
Distribution and quantity of endophytic fungal genera in different tissues of the sweetpotato variety ‘Guangcaishu 16–19’. (**A**–**F**): leaf (**A**), petiole (**B**), tender stem (**C**), old stem (**D**), root (**E**), and Venn diagram (**F**). TS, tender stem; OS, old stem.

At the genus level, *Colletotrichum* was the dominant endophytic fungus in ‘Guangcaishu 16–19’, followed by *Fusarium*, and both genera were widely distributed across different tissues. The number of genera isolated from each tissue was as follows: leaf ≥ tender stem > petiole > old stem ≥ root. *Amphirosellinia* and *Xylaria* were exclusively distributed in leaves and petioles. *Hypoxylon*, *Muyocopron*, *Phyllosticta*, and *Psathyrella* were only found on the leaf blades. *Aspergillus* was only isolated from tender and old stems, whereas *Chaetomium* was detected only on the tender stems and roots. In addition to the dominant genera, *Trichoderma* and *Diaporthe* were also the dominant genera in roots. The relative isolation frequencies of the other fungal genera in each tissue were all less than 10%, which further indicated that the dominant genera were widely distributed across the tissues and held a dominant position (Figure 6).

**Figure 7:**
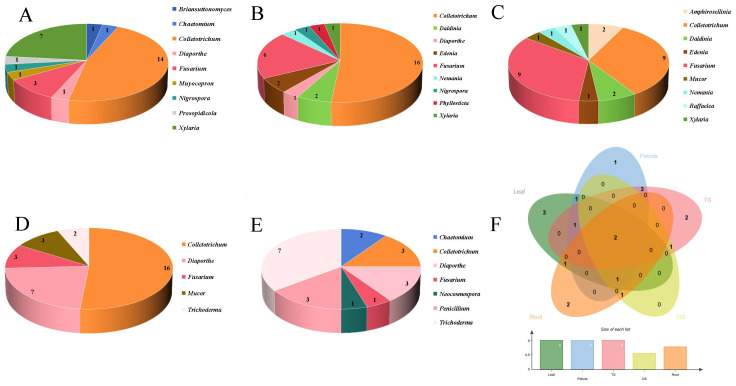
Distribution and quantity of the endophytic fungal genera in different tissues of the sweetpotato variety ‘Guangcaishu No. 5’. (**A**–**F**): leaf (**A**), petiole (**B**), tender stem (**C**), old stem (**D**), root (**E**) and Venn diagram (**F**).

At the genus level, *Colletotrichum* was the dominant endophytic fungus in ‘Guangcaishu No. 5’, followed by *Fusarium*, and both genera were widely distributed across different tissues. The number of genera isolated from each tissue was as follows: leaf ≥ petiole ≥ tender stem > root > old stem. The dominant genera shared between the old stems and roots were *Colletotrichum*, *Diaporthe*, and *Trichoderma*. *Phyllosticta* was found exclusively in the petioles. *Briansuttonomyces*, *Chaetomium*, *Muyocopron*, and *Prosopidicola* were isolated solely from leaves. *Amphirosellinia*, *Mucor*, and *Raffaelea* were isolated exclusively from the tender stems, whereas *Neocosmospora* and *Penicillium* were only detected in the roots. In addition to the dominant genera, the endophytes associated with roots included *Trichoderma* and *Penicillium*, and *Trichoderma* was isolated the most frequently (Figure 7).

**Figure 8:**
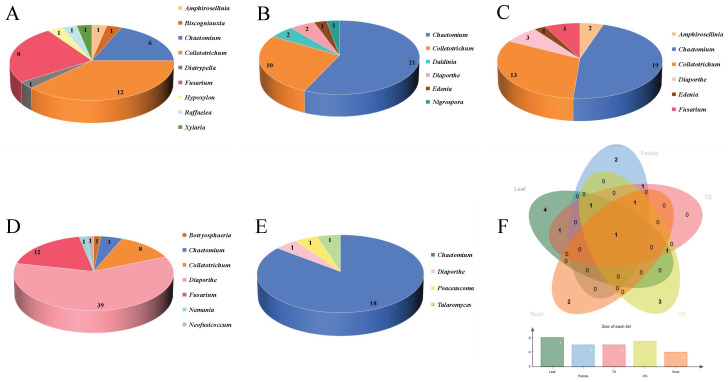
Distribution and quantity of the endophytic fungal genera in different tissues of the sweetpotato variety ‘Guangshu 87’. (**A**–**F**): leaf (**A**), petiole (**B**), tender stem (**C**), old stem (**D**), root (**E**), and Venn diagram (**F**).

At the genus level, *Chaetomium* was the dominant endophytic fungus in ‘Guangshu 87’ and was widely distributed across all the tissues. *Colletotrichum* was the second most prevalent genus, although it was not isolated from the roots. The number of genera isolated from each tissue was as follows: leaf > old stem > petiole ≥ tender stem > root. Notably, *Diaporthe* was the most frequently isolated genus in the old stems in which it was highly enriched. Additionally, *Colletotrichum* was the dominant genus in the leaves. *Raffaelea* and *Xylaria* were detected exclusively in the leaves, whereas *Daldinia* and *Nigrospora* were found only in the petioles. The genera specific to the old stems included *Botryosphaeria*, *Nemania*, and *Neofusicoccum*, while *Poaceascoma* and *Talaromyces* were detected only in the roots (Figure 8).

As shown in the figures, *Colletotrichum* was the dominant genus shared by all three varieties. Except for the roots of ‘Guangshu 87’, *Colletotrichum* was widely distributed across all the tissues of the three varieties, although its isolation frequency was lowest in the roots of each variety. *Fusarium* was the secondary dominant genus in the leafy varieties and also broadly distributed among the different tissues. However, it occurred less frequently in the petioles and roots. The dominant genus for the tuberous variety was *Chaetomium*, followed by *Colletotrichum* and *Diaporthe*. Overall, some fungal genera were widely distributed across multiple tissues and showed low tissue specificity, whereas others were confined to one or a few tissues, indicating possible tissue preference.

### 3.5. Distribution of Endophytic Fungi in Different Tissues of Three Sweetpotato Varieties at the ITS-Based Putative Taxon Level

**Figure 9:**
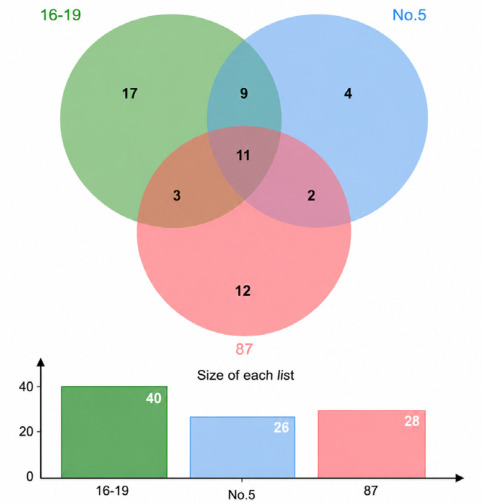
Venn diagram of ITS-based putative endophytic fungal taxa among three sweetpotato varieties.

**Figure 10:**
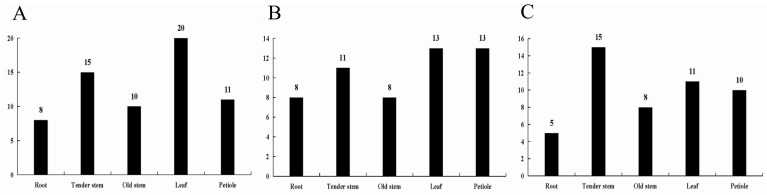
Number of ITS-based putative endophytic fungal taxa isolated from different tissues of three sweetpotato varieties. (**A**–**C**): ‘Guangcaishu 16–19’ (**A**), ‘Guangcaishu No. 5’ (**B**) and ‘Guangshu 87’ (**C**).

A Venn analysis revealed the following trend in the number of isolated endophytic fungi at the ITS-based putative taxon level: ‘Guangcaishu 16–19’ > ‘Guangshu 87’ > ‘Guangcaishu No. 5’. A total of 11 putative taxa were shared among all three varieties. Additionally, ‘Guangcaishu 16–19’ and ‘Guangcaishu No. 5’ shared nine putative taxa, while they shared only three and two putative taxa with ‘Guangshu 87’, respectively (Figure 9). The distribution of different putative taxa varied among tissues and varieties (Figure 10).

Among the ITS-based putative taxa, isolates assigned to *Colletotrichum brevisporum* were shared by all three varieties, although they were not detected in the roots of ‘Guangshu 87’. Isolates assigned to *Colletotrichum chlorophyti* and *Fusarium pseudoanthophilum* were predominant in the leafy sweetpotato varieties, whereas isolates assigned to *Chaetomium spirochaete* and *Diaporthe batatas* were predominant in the tuberous sweetpotato variety.

**Figure 11:**
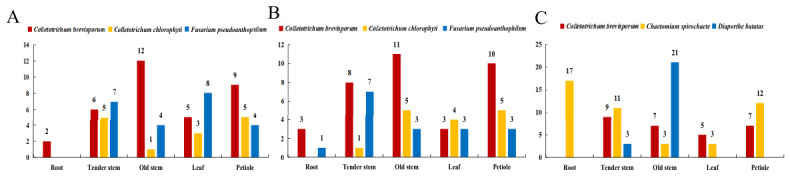
Number of endophytic fungal isolates assigned to ITS-based putative taxa in different tissues of three sweetpotato varieties. (**A**–**C**): ‘Guangcaishu 16–19’ (**A**), ‘Guangcaishu No. 5’ (**B**) and ‘Guangshu 87’ (**C**).

In ‘Guangcaishu 16–19’, isolates assigned to *Colletotrichum brevisporum* were detected in all tissues and were most abundant in the old stems. Isolates assigned to *Colletotrichum chlorophyti* and *Fusarium pseudoanthophilum* were detected in all tissues except for roots (Figure 11A). In ‘Guangcaishu No. 5’, isolates assigned to *Colletotrichum brevisporum* and *Fusarium pseudoanthophilum* were detected in all tissues, whereas isolates assigned to *Colletotrichum chlorophyti* were not detected in the roots (Figure 11B). In ‘Guangshu 87’, isolates assigned to *Chaetomium spirochaete* were detected in all tissues, whereas isolates assigned to *Colletotrichum brevisporum* were not detected in the roots. Isolates assigned to *Diaporthe batatas* were predominantly detected in the tender and old stems and were most abundant in the old stems (Figure 11C).

At the ITS-based putative taxon level, isolates assigned to *Chaetomium spirochaete*, *Colletotrichum brevisporum*, *Colletotrichum chlorophyti*, *Diaporthe batatas*, and *Fusarium pseudoanthophilum* were commonly detected among the three sweetpotato varieties. In contrast, some putative taxa showed clear variety-specific distribution patterns. For example, isolates assigned to *Aspergillus assiutensis* and *Aspergillus flavus* were detected only in ‘Guangcaishu 16–19’. In addition, the relative isolation frequencies of the same putative taxa differed between the leafy and tuberous sweetpotato varieties. For instance, isolates assigned to *Chaetomium spirochaete* were detected in the leafy varieties at relative isolation frequencies of 4.76% and 0.75%, respectively, whereas their relative isolation frequency reached 24.08% in the tuberous variety. Similarly, isolates assigned to *Diaporthe batatas* showed a substantially higher relative isolation frequency in the tuberous variety than in the two leafy varieties.

### 3.6. Comparative Study on the Diversity of Endophytic Fungi in Sweetpotatoes

The diversity of endophytic fungi was assessed using the Shannon–Wiener index (H), Simpson’s index (D), and Pielou’s evenness index (J). The alpha diversity followed the pattern ‘Guangcaishu 16–19’ > ‘Guangcaishu No. 5’ > ‘Guangshu 87’. ‘Guangcaishu 16–19’ exhibited the highest alpha diversity, indicating that its endophytic fungal community had greater richness and was more evenly distributed (Table 2).

In the two leafy sweetpotato varieties, the roots showed the highest values of the Shannon–Wiener index, Simpson’s diversity index, and Pielou’s evenness index. In ‘Guangcaishu 16–19’, the petioles exhibited the lowest values for all three diversity indices. In ‘Guangcaishu No. 5’, the petioles had the lowest Pielou’s evenness index, whereas the old stems showed the lowest Shannon–Wiener and Simpson’s diversity indices. In the tuberous sweetpotato variety Guangshu 87, the leaves had the highest values for all three diversity indices, while the roots showed the lowest values (Table 3).

Similarity coefficients were used to assess the similarity of endophytic fungal communities among the three sweetpotato varieties, with values ranging from 0.50 to 0.67. The highest similarity (0.67) was observed between ‘Guangcaishu 16–19’ and ‘Guangcaishu No. 5’ followed by ‘Guangcaishu 16–19’ and ‘Guangshu 87’ (0.55), while the lowest similarity (0.50) was found between ‘Guangshu 87’ and ‘Guangcaishu No. 5’ (Table 4).

The highest similarity coefficient (0.84) was observed between the petioles of ‘Guangcaishu 16–19’ and the tender stems of ‘Guangcaishu No. 5’, with eight shared genera. A coefficient of 0.77 was observed between the roots of ‘Guangcaishu 16–19’ and the old stems of ‘Guangcaishu No. 5’, as well as between the petioles and tender stems of ‘Guangshu 87’. Each had five shared genera (Table 5).

## 4. Discussion

Sweetpotatoes, an important food and cash crop, are rich in diverse nutritional components and bioactive compounds. This diversity also extends to their microbiome. The alpha diversity analysis of three varieties of sweetpotato in this study revealed that the Shannon–Wiener index (2.48–2.96), Simpson’s index (0.88–0.91), and Pielou’s evenness index (0.74–0.81) were all at relatively high levels. This indicates that the endophytic fungal communities within sweetpotatoes had high levels of alpha diversity. This finding is consistent with the results from related studies on other plants [30].

The alpha diversity of endophytic fungal communities in the roots of the leafy sweetpotato varieties ‘Guangcaishu 16–19’ and ‘Guangcaishu No. 5’ was higher than that observed in the tuberous variety ‘Guangshu 87’. This difference may be associated with differences in growth strategies among different types of sweetpotato varieties. The leafy varieties prioritize the development of aboveground tissues, which may influence the recruitment of rhizosphere microbes. Their root exudates create a more permissive microenvironment, thereby supporting a more diverse fungal community [31,32,33]. In contrast, the rhizosphere microenvironment of the tuberous varieties may be more conducive to selecting microbial communities specialized in promoting the enlargement of tubers and storing carbon and N [34,35].

*Colletotrichum* was widely distributed across three varieties. Beyond this core group, variations exist among the different varieties. *Fusarium* was another dominant genus in the leafy sweetpotato varieties, whereas *Chaetomium* predominated in the tuberous variety. These fungal genera are also extensively present in plants across other regions [36,37,38]. The variation in dominant fungal genera among the varieties be influenced by the host genotype [39]. In addition to host genotype, differences in root exudation patterns and plant immune responses may also shape the recruitment and assembly of endophytic fungal communities.

This study identified *Mucor*, *Muyocopron*, *Phyllosticta*, and *Trichoderma* as genera specific to the leafy sweetpotato varieties since they were absent in ‘Guangshu 87’, thus indicating host preference in the colonization by endophytic fungi [40]. A similarity analysis further showed that the fungal communities of the two leafy varieties were highly similar, whereas their community structure was distinct from that of the tuberous variety ‘Guangshu 87’. However, since only one tuberous variety was included in this study, these findings primarily reflect the differences between leafy varieties and this specific tuber variety, and thus, cannot be generalized to all the tuberous sweetpotato varieties. Nevertheless, biological traits related to the use of sweetpotato varieties, specifically different domestication selections for stems and leaves versus tubers, may influence the composition of endophytic fungal communities [41,42]. This hypothesis warrants further validation using a larger number of tuberous and leafy varieties in future studies.

The endophytic fungi communities in the three varieties of sweetpotato at the genus level follow a pattern of “leaf and stem > root.” The rhizosphere serves as the primary entry point connecting plants to the vast reservoir of soil microorganisms, where microbial diversity and abundance are typically highest [43]. This apparently contradictory pattern may be explained by selective filtering within the plant and differences in colonization mechanisms among tissues. Although roots are exposed to a diverse pool of microorganisms, the plant establishes stringent physical barriers in the roots, such as the endodermis and immune defenses, creating a “screening bottleneck” that allows only a limited number of well-adapted strains to colonize as endophytes [44]. In contrast, leaves represent more open environments, providing direct entry routes for airborne microorganisms through natural openings such as stomata [45].

The endophytic fungal alpha diversity of the scab-resistant variety ‘Guangcaishu 16–19’ was higher than that of the susceptible variety ‘Guangcaishu No. 5’. This finding is consistent with our previous high-throughput sequencing study that compared healthy ‘Guangcaishu 16–19’ with scab-infected ‘Guangcaishu No. 5’ [46]. The higher endophytic fungal diversity in ‘Guangcaishu 16–19’ may endow it with a richer reservoir of potentially beneficial functions, thereby enhancing its disease resistance through mechanisms such as the activation of systemic resistance [47], competition for ecological niches, or the production of antimicrobial compounds [48]. Although the fungal community compositions of the two varieties are highly similar, there were marked differences in their resistance to disease. This suggests that resistance to scab disease may depend primarily on the presence or relative abundance of specific beneficial endophytic fungal groups, rather than the overall similarity of the community composition [49]. However, this possible association between specific endophytic fungal groups and scab resistance requires further validation. In future studies, representative isolates, particularly those enriched in the scab-resistant variety or belonging to potentially beneficial fungal groups, should be identified to the species level using multilocus phylogenetic analyses, and their interactions with sweetpotato scab pathogens should be assessed to clarify their potential contribution to scab resistance.

Several specific fungal taxa were detected in the three varieties of sweetpotato and their different tissues. This phenomenon may be driven by the joint influence of host genetic regulation and tissue microenvironment ecological filtering. On the one hand, the genotypes of different varieties shape distinct chemical environments by regulating the composition of root exudates and the intensity of immune responses, thereby exerting differential selective pressures on the fungi attempting to colonize the tissue [50,51]. Alternatively, diverse tissues, such as roots, stems, and leaves, provide distinct ecological niches for fungal colonization owing to differences in their physicochemical properties, resulting in the selective recruitment of microbial communities from the surrounding environment [52].

Endophytic fungi serve as crucial symbionts for plants and have indispensable roles in aiding hosts against pests and diseases, promoting growth and the uptake of nutrients, and enhancing resistance to abiotic stresses [53,54]. Simultaneously, they are significant sources of bioactive compounds and capable of producing diverse structurally novel secondary metabolites [55]. The endophytic fungi isolated in this study can be functionally categorized into four groups: potentially pathogenic genera, biocontrol genera, functionally dual-purpose genera, and genera that are taxonomically novel and understudied or lack unclear ecological functions.

*Chaetomium* has a promising biocontrol potential because it can control the diseases through multiple mechanisms, such as by the production of antimicrobial compounds, competition for nutrients, and the induction of systemic plant resistance, while also promoting plant growth [56]. *Trichoderma* species, as widely utilized beneficial fungi, not only produce a variety of enzymes with antimicrobial activity [57], but also generate secondary metabolites, such as trichodermin and gliovirin, that exhibit significant antagonistic activity against pathogenic fungi [58,59]. This study also isolated several potential fungal pathogens. For example, species of *Colletotrichum* can cause sweetpotato anthracnose [60] and are also the causal agent of potato black rot disease [61], as well as anthracnose and soft rot in loquat fruits [62]. Similarly, *Fusarium* is a major causal agent of root rot in sweetpotato [63] and is responsible for severe diseases, such as *Fusarium* wilt of banana (*Musa acuminata*) [64], which can lead to significant losses in yield. Notably, these pathogens can exist as endophytes in healthy plants without causing visible symptoms. Elucidating the mechanisms underlying their transition from an endophytic to a pathogenic lifestyle will therefore be crucial for future disease management strategies [65,66]. Additionally, some fungal strains isolated in this study had dual functions. For example, the genus *Penicillium* encompasses pathogens, such as *P. expansum* [67], while also including beneficial species, such as *P. notatum* and *P. chrysogenum*. The diverse secondary metabolites produced by these beneficial species, such as antibiotics and organic acids, are of significant value [68].

Some endophytic fungi isolated in culture cannot be identified by morphology alone because they do not sporulate; therefore, this study used ITS sequencing, a standard DNA barcoding technique, for their identification [69]. Although ITS sequence analysis generally provides reliable genus-level assignments for fungal isolates, its resolution may be insufficient for species-level identification in some fungal groups. Therefore, species-level assignments in this study should be regarded as ITS-based putative taxa and confirmed by multilocus phylogenetic analyses using additional markers, such as *TEF1-α*, *calmodulin*, *β-tubulin*, and LSU [70]. Additionally, this study has some limitations. Although pesticide application was uniform across all experimental plots, its potential influence on endophytic fungal communities cannot be completely excluded. Factors, such as the type of culture medium and the duration of the surface disinfection of plant tissue, exert an influence on the species and quantity of endophytic fungi isolated. Increasing the number of sampled plant varieties or tissues would more accurately reflect the diversity and distribution of the endophytic fungi within plants. Geographical location and climatic conditions also influence the diversity of endophytic fungi within plants. Given the difficulty in culturing certain endophytic fungi in vitro, high-throughput sequencing methods can be utilized for more comprehensive analyses. These factors should be fully considered in subsequent research.

Future research will focus on representative isolates from genera with reported biocontrol potential or from fungal groups enriched in the scab-resistant variety, with the aim of evaluating their antagonistic activity, plant-growth-promoting ability, and stress tolerance, characterizing their bioactive secondary metabolites, and elucidating the corresponding biosynthetic pathways, thereby expanding their potential applications in green pesticides and sustainable agriculture.

## 5. Conclusions

This study analyzed the diversity, community composition, and tissue distribution of culturable endophytic fungi in two leafy sweetpotato varieties with contrasting responses to scab disease and one tuberous sweetpotato variety. Based on ITS sequence analysis, 492 isolates were assigned to 63 putative taxa in 31 genera. Community composition varied among varieties and tissues: *Colletotrichum* was dominant in the two leafy varieties, whereas *Chaetomium* predominated in the tuberous variety; overall, genus-level richness and isolate abundance were higher in leaves and stems than in roots. The two leafy varieties showed the greatest community similarity, and their root-associated communities exhibited higher alpha diversity than that of the tuberous variety. In addition, the scab-resistant variety harbored higher endophytic fungal diversity than the susceptible variety, suggesting a possible association between endophytic fungal diversity and scab resistance. These findings provide a culturable fungal resource for future screening of beneficial endophytic fungi, bioactive metabolites, and potential biocontrol agents. However, multilocus phylogenetic analyses using additional markers are still needed to confirm species-level identifications and support subsequent functional evaluation of these fungal resources.

## Figures and Tables

**Table 1:** Taxonomic classification and isolation frequency of endophytic fungi identified based on morphological characteristics.

Phylum	Class	Order	Family	Genus	Number	IF (%)
Ascomycota	Dothideomycetes	Botryosphaeriales	Botryosphaeriaceae	*Botryosphaeria*	1	0.23
	Eurotiomycetes	Eurotiales	Aspergillaceae	*Aspergillus*	3	0.69
				*Penicillium*	4	0.92
	Sordariomycetes	Amphisphaeriales	Apiosporaceae	*Nigrospora*	3	0.69
		Diaporthales	Diaporthaceae	*Diaporthe*	64	14.68
		Glomerellales	Glomerellaceae	*Colletotrichum*	165	37.84
		Hypocreales	Hypocreaceae	*Trichoderma*	18	4.13
			Nectriaceae	*Fusarium*	78	17.89
		Sordariales	Chaetomiaceae	*Acrophialophora*	1	0.23
				*Chaetomium*	73	16.74
		Xylariales	Hypoxylaceae	*Daldinia*	12	2.75
				*Hypoxylon*	2	0.46
			Xylariaceae	*Xylaria*	12	2.75

Note: IF (%) = *N_i_*/N × 100, where *N_i_* is the number of isolates in each genus and N is the total number of morphologically identified isolates, N = 436.

**Table 2:** Diversity indices of endophytic fungi and the number of ITS-identified isolates from three sweetpotato varieties.

	Shannon-Wiener Index (*H*)	Simpson Diversity Index (*D*)	Pielou’s Evenness Index (*J*)	No. of Isolates (*N*)
‘Guangcaishu 16–19’	2.96	0.91	0.81	157
‘Guangcaishu No. 5’	2.72	0.89	0.79	139
‘Guangshu 87’	2.48	0.88	0.74	196

Note: Values in the “No. of isolates (*N*)” column indicate the number of ITS-identified isolates (total *N* = 492).

**Table 3:** Diversity indices of endophytic fungi in different tissues of three sweetpotato varieties.

	Shannon-Wiener			Simpson Diversity Index			Pielou’s Evenness Index		
	Guangcaishu 16–19	Guangcaishu No. 5	Guangshu87	Guangcaishu 16–19	Guangcaishu No. 5	Guangshu87	Guangcaishu 16–19	Guangcaishu No. 5	Guangshu87
Leaf	1.65	1.61	1.68	0.71	0.71	0.76	0.72	0.73	0.76
Petiole	1.43	1.57	1.19	0.66	0.68	0.60	0.65	0.71	0.66
Tender stem	1.69	1.72	1.34	0.73	0.76	0.67	0.73	0.78	0.75
Old stem	1.66	1.31	1.21	0.75	0.66	0.59	0.80	0.81	0.62
Root	1.84	1.75	0.73	0.81	0.80	0.32	0.88	0.90	0.45

**Table 4:** Similarity coefficients of the endophytic fungal communities among three sweetpotato varieties.

Varieties	‘Guangshu 87’	‘Guangcaishu No. 5’	‘Guangcaishu 16–19’
‘Guangshu 87’	---	0.50	---
‘Guangcaishu 16–19’	0.55	---	---
‘Guangcaishu No. 5’	---	---	0.67

Note: ---, no similarity coefficient.

**Table 5:** Similarity matrix of endophytic fungal communities across tissue samples from three sweetpotato varieties.

	1	2	3	4	5	6	7	8	9	10	11	12	13	14	15
1	1.00														
2	0.67	1.00													
3	0.40	0.50	1.00												
4	0.33	0.44	0.56	1.00											
5	0.33	0.44	0.67	0.50	1.00										
6	0.42	0.42	0.32	0.35	0.47	1.00									
7	0.53	0.74	0.53	0.47	0.47	0.56	1.00								
8	0.63	0.84	0.63	0.47	0.47	0.33	0.67	1.00							
9	0.27	0.40	0.53	0.62	0.77	0.43	0.29	0.43	1.00						
10	0.24	0.35	0.59	0.40	0.67	0.50	0.38	0.25	0.67	1.00					
11	0.53	0.53	0.32	0.24	0.35	0.44	0.33	0.56	0.29	0.38	1.00				
12	0.35	0.59	0.59	0.40	0.67	0.63	0.75	0.50	0.50	0.57	0.38	1.00			
13	0.38	0.63	0.50	0.43	0.57	0.53	0.53	0.53	0.55	0.62	0.53	0.77	1.00		
14	0.35	0.47	0.47	0.53	0.53	0.50	0.50	0.38	0.50	0.57	0.38	0.57	0.62	1.00	
15	0.00	0.14	0.14	0.17	0.33	0.31	0.15	0.00	0.22	0.36	0.15	0.36	0.40	0.36	1.00

Note: Values represent pairwise similarity coefficients of endophytic fungal communities among samples 1–15. Diagonal values are 1.00 and indicate self-comparisons. Because the matrix is symmetric, the upper triangle is omitted to avoid duplicate values. Samples 1–5 represent the leaves, petioles, tender stems, old stems, and roots of ‘Guangcaishu 16–19’, respectively; samples 6–10 represent the corresponding tissues of ‘Guangcaishu No. 5’; and samples 11–15 represent the corresponding tissues of ‘Guangshu 87’.

## Data Availability

The original contributions presented in this study are included in the article/Supplementary Materials. Further inquiries can be directed to the corresponding author.

## Supplementary Materials

The following supporting information can be downloaded at: https://www.mdpi.com/article/10.3390/jof12060394/s1, Table S1. NCBI accession numbers of endophytic fungal isolates obtained in this study.
